# Evidence of intermetastatic heterogeneity for pathological response and genetic mutations within colorectal liver metastases following preoperative chemotherapy

**DOI:** 10.18632/oncotarget.7809

**Published:** 2016-03-01

**Authors:** Mylène Sebagh, Marc-Antoine Allard, Nelly Bosselut, Myriam Dao, Eric Vibert, Maïté Lewin, Antoinette Lemoine, Daniel Cherqui, René Adam, Antonio Sa Cunha

**Affiliations:** ^1^ AP-HP Hôpital Paul Brousse, Department of Pathology, Villejuif, France; ^2^ Inserm U1193, Paris-Sud University, Villejuif, France; ^3^ AP-HP Hôpital Paul Brousse, Hepatobiliary Centre, Villejuif, France; ^4^ Inserm U935, Paris-Sud University, Villejuif, France; ^5^ AP-HP Hôpital Paul Brousse, Department of Oncogenetics, Villejuif, France; ^6^ AP-HP Hôpital Paul Brousse, Radiology, Villejuif, France

**Keywords:** colorectal liver metastases, pathological response, intermetastatic tumor heterogeneity, somatic gene profile

## Abstract

**Background:**

In patients receiving preoperative chemotherapy, colorectal liver metastases (CLM) are expected to demonstrate a similar behaviour because of similar organ microenvironment and tumour cell chemosensitivity. We focused on the occurrence of pathological and genetic heterogeneity within CLM.

**Methods:**

Patients resected for multiple CLM between 2004 and 2011 after > three cycles of chemotherapy were included. Pathological heterogeneity was arbitrarily defined as a > 50% difference in the percentage of remaining tumour cells between individual CLM. In patients with pathological heterogeneity, the mutational genotyping (KRAS, NRAS, BRAF and PIK3CA) was determined from the most heterogeneous CLM.

**Results:**

Pathological heterogeneity was observed in 31 of 157 patients with multiple CLM (median = 4, range, 2–32) (19.7%). In 72.4% of them, we found a concordance of the mutation status between the paired CLM: both wild-type in 55%, and both mutated in 17.2%. We observed a discordance of the mutation status of 27.6% between CLM: one mutated and the other wild-type. The mutated CLM was the less florid one in 75% of patients with genetic heterogeneity.

**Conclusions:**

Pathological heterogeneity is present in 19.7% of patients with multiple CLM. Genetic heterogeneity is present in 27.6% of patients with pathological heterogeneity. Heterogeneity could refine guide management for tissue sampling.

## INTRODUCTION

Preoperative chemotherapy is an important part of the management of patients with colorectal liver metastases (CLM). Response evaluation is usually assessed on a radiological basis using the RECIST criteria [1]. Response is determined as the total change in sum of diameters of all pre-defined target lesions following chemotherapy. The RECIST criteria do not take account individual lesion response. Recently, Van Kessel et al. showed a radiological heterogeneity in approximately 35% of patients with CLM, suggesting underlying pathological heterogeneity [2]. The pathological response (PR) has been validated as a morphologic variable to assess response to chemotherapy. To date, the PR is assessed by four methods based on the presence of viable cancer cells, which however differed in patients with multiple CLM [3–6]. CLM are expected to demonstrate a similar behaviour since the organ microenvironment and tumour cell chemosensitivity were thought similar. However, no study has addressed the intermetastatic behaviour on a pathological level.

Information not only on *KRAS* but also in *NRAS* mutational status is now an essential prerequisite for the selection of a targeted therapy based on anti-EGFR therapies. Moreover, mutations in *KRAS* and other genes such as *BRAF* and *PIK3CA* are also associated with worse survival [7]. Variations in genetic alterations between primary colorectal cancer and metastatic lesions of different sites in the same patient have been extensively reported. No study has investigated the incidence and the impact of intermetastatic heterogeneity on a genetic level within CLM.

This study aimed to assess the occurrence of pathological heterogeneity in patients with multiple CLM and its predictive factors, and the relationship between genetic and pathological heterogeneity.

## RESULTS

### Patient characteristics

The study included 157 patients with multiple CLM. Of them, 87 patients (55%) received oxaliplatin-based chemotherapy, and 70 patients (45%) received irinotecan-based chemotherapy. Sixty patients (38%) received bevacizumab, and 26 patients (16.5%) received cetuximab. The median number of chemotherapy cycles was six (range, 3–34). The mean and median numbers of CLM were 4.68 (+ 3.6) and 4 (range, 2 to 32), respectively. The mean and median maximum tumour size were 3.3 cm (+ 2.2 cm) and 3 cm (range: 0.1–11 cm), respectively. One hundred and six patients (67%) had positive resection margins on pathological examination of the specimen (R1 resection).

### Pathological heterogeneity and associated factors

Seven patients had a complete response. The remaining 150 patients were classified as follows: according to the method by Blazer et al. [4], 83 patients had a major and 67 patients a minor tumour response. According to the method by Sebagh et al. [6], 55 patients had < 6 cm-residual tumour and 95 patients had > 6 cm-residual tumour. There was no difference in the mean PR between the 86 patients treated by chemotherapy plus biotherapy and the 71 patients treated by chemotherapy alone (38.9% versus 41.9% according to the method by Blazer).

The mean and median difference in the PR between the most heterogeneous CLM were 30.4% (+ SD = 29.6%) and 25% (range: 0–100%), respectively. Pathological homogeneity was observed in 126 patients (80.2%) including the 7 patients with complete response. Pathological heterogeneity of > 50% was observed in 31 patients (19.7%).

Pathological heterogeneity of > 50% was significantly associated with none of the clinical and pathological variables (Table 1). The mean difference in the PR was significantly higher in patients with > 3 CLM (37.1% vs 13.8%, *p* < 0.0001) and in patients undergoing preoperative portal vein embolization (PVE) (37.9% vs 27.2%, *p* = 0.05), tended to be higher in patients with a number of preoperative chemotherapy cycles > 6 (*p* = 0.07) and was not impacted by the global PR, nor the addition of targeted therapies under univariate analysis (Table 2). The multivariate analysis identified a number of CLM > 3 and the use of preoperative PVE as independent factors (*p* = 0.002 and 0.04, respectively).

**Table 1:** Univariate analysis of factors associated with pathological heterogeneity of more than 50%

Variables		Absent		Present		*P*
		No.	%	No.	%	
Age	≤ 65 years	81	64.3	24	77.4	0.24
	> 65 years	45	35.7	7	22.6	
Gender	Female	53	42.1	12	38.7	0.89
	Male	73	57.9	19	61.3	
**Liver disease**						
Synchronous	N	30	23.8	5	16.1	0.49
	Y	96	76.2	26	83.9	
Tumor location	Unilobar	33	26.2	10	32.3	0.65
	Bilobar	93	73.8	21	67.7	
No. Tumor	2	40	31.7	5	16.1	0.13
	> 2	86	68.3	26	83.9	
No. Tumor	2 to 3	63	50.0	10	32.3	0.12
	> 3	63	50.0	21	67.7	
No. Tumor	2 to 4	82	65.1	17	54.8	0.40
	> 4	44	34.9	14	45.2	
No. Tumor	2 to 5	98	77.8	20	64.5	0.19
	> 5	28	22.2	11	35.5	
**Preoperative management**						
Portal vein embolization	N	93	73.8	17	54.8	**0.06**
	Y	33	26.2	14	45.2	
Biotherapy	N	59	46.8	12	38.7	0.54
	Y	67	53.2	19	61.3	
Cetuximab	N	105	83.3	26	83.9	1.00
	Y	21	16.7	5	16.1	
Bevacizumab	N	80	63.5	17	54.8	0.50
	Y	46	36.5	14	45.2	
Oxaliplatin	N	53	42.1	11	35.5	0.64
	Y	73	57.9	20	64.5	
No. Preop cycles	3 to 6	73	57.9	12	38.7	0.08
	> 6	53	42.1	19	61.3	
No. Preop cycles	3 to 8	89	70.6	18	58.1	0.26
	> 8	37	29.4	13	41.9	
No. Preop cycles	3 to 10	104	82.5	23	74.2	0.42
	> 10	22	17.5	8	25.8	
**Pathological data**						
Difference in tumor size	0 to 2 cm	70	55.6	18	58.1	0.96
	> 2 cm	56	44.4	13	41.9	
Pathological response						
Blazzer et al	> 50%	52	41.3	15	48.4	0.61
(mean % of residual tumor cells)	< 50%	74	58.7	16	51.6	
Sebagh et al	> 6	78	61.9	17	54.8	0.61
(cm-residual tumor)	< 6	48	38.1	14	45.2	

**Table 2:** Univariate and multivariate analysis for the mean difference in the pathological response (between the CLM with the highest and the lowest response)

Variables		Mean	SD	*P*	Multivariate analysis	
					Estimate	*P*
Age	≤ 65 years	31.3	31	0.59		
	> 65 years	28.7	26			
Gender	Female	30.2	30	0.94		
	Male	30.6	29			
**Liver disease**						
Synchronous	N	25.7	28	0.29		
	Y	31.6	30			
Tumor location	Unilobar	32.7	31	0.56		
	Bilobar	29.6	29			
No. Tumor	2	13.8	24	< 0.0001	14.4	0.002
	> 2	37.1	29			
No. Tumor	2 to 3	21.9	27	0.0006		
	> 3	37.8	30			
No. Tumor	2 to 4	25.4	29	0.006		
	> 4	38.9	29			
No. Tumor	2 to 5	27.2	29	0.02		
	> 5	40.1	31			
**Preoperative management**						
Portal vein embolization	N	27.2	28	0.05	10.2	**0.04**
	Y	37.9	33			
Biotherapy	N	29.2	27	0.64		
	Y	31.4	32			
Cetuximab	N	30.4	29	0.98		
	Y	30.6	31			
Bevacizumab	N	29.6	28	0.66		
	Y	31.8	32			
Oxaliplatin	N	31.5	30	0.72		
	Y	29.7	29			
No. Preop cycle	3 to 6	26.5	27	0.07		
	> 6	35.1	32			
No. Preop cycle	3 to 8	28.5	29	0.24		
	> 8	34.5	30			
No. Preop cycle	3 to 10	30.5	29	0.91		
	> 10	29.1	33			
**Pathological data**						
Difference in tumor size	0 to 2 cm	29.2	32	0.55		
	> 2 cm	32.0	27			
Global pathologic response						
Blazzer et al	> 50%	34.5	29	0.14		
(mean % of residual tumor cells)	< 50%	27.4	30			
Sebagh et al	0 to 6	29.0	33	0.64		
(cm-residual tumor)	> 6	31.3	27			

SD, Standard deviation; Intercept = 23.10, R2 = 0.1

### Gene mutation status (Table 3)

**Table 3:** Gene somatic profile in the overall population

	N	*KRAS*	*NRAS*	*BRAF*	*PIKCA3*	*KRAS + PIKCA3*	
		exon 2/3/4	exon 2/3/4	exon 11/15	exon 9/20	exon 2	exon 9/20
**Patients without pathological heterogeneity**	126						
ND (Complete response)	7	–	–	–	–	–	
Mutation	42	33 (31/1/1)	3 (3/0/0)	1 (0/1)	3 (2/1)	2 (1/1)	
No mutation	77	0	0	0	0	0	
**Patients with pathological heterogeneity**	31						
**Two samples**	29						
No mutation within both CLM	16	0	0	0	0	0	
Mutation within both CLM	5	3 (2/0/1)	1 (2/0/0)	0	0	1 (1/0)	
Mutation within one of both CLM	8	5 (5/0/0)	0	2 (2/0)	0	1 (1/0)	
**One sample**	2						
No mutation	1*	0	0	0	0	0	
Mutation	1**	1 (1/0/0)	0	0	0	0	
ND, not done							

* complete remission of the second CLM.

** non amplified DNA of the second CLM.

In the overall population, a 30.6% rate of *KRAS* mutations, 2% rate of *BRAF* mutations, 2.7% rate of *NRAS* mutations and 4.7% rate of *PIK3CA* mutations were observed. In total, 37.3% of patients showed at least one mutation. The frequency of gene mutation was not significantly different between the patients with and without pathological heterogeneity (14/31 versus 42/119).

In the patients without pathological heterogeneity (*N* = 126), the mutational status was necessarily unavailable in the 7 patients with complete response. of the remaining 119 patients, 77 patients did not exhibit mutations and 42 patients (35.2%) had one or more mutations: 35 patients had *KRAS* mutations, 3 *NRAS* mutations, 1 *BRAF* mutation and 5 *PIK3CA* mutations, associated with *KRAS* mutations in 2.

In the patients with pathological heterogeneity (*N* = 31), genotyping was available from the two most heterogeneous CLM in 29 patients. In the remaining 2 patients, the mutational status was available in only one CLM: In the first patient having 2 CLM, one CLM was not tested because of a complete response and the second CLM did not exhibit mutation. In the second patient, one CLM exhibited a *KRAS* mutation and the DNA from the second CLM was unamplified.

In the 29 patients with available genotyping from the two most heterogeneous CLM, 13 patients had at least one mutation within at least one CLM: eight patients had *KRAS* mutations, one had *NRAS* mutation, two had *BRAF* mutation and two had concurrent *PIK3CA* and *KRAS* mutations. Table 4 showed that both tested CLM were mutated in five patients. The same mutational status was present within each CLM in 5/5 patients (100%). Both CLM were wild-type in 16 patients. One mutated CLM and one wild-type CLM were present in eight patients, giving a genetic heterogeneity of 27.6% of the patients with pathological heterogeneity. In 6 of these 8 patients, the mutated CLM was the less florid.

**Table 4:** Mutation status of the most heterogeneous CLM in the 31 patients with pathological heterogeneity

		CLM N°1			CLM N°2		
Patients N°	%*	Mutation Status**	Type of mutation	%* response (%)	Mutation Status**	Type of mutation	Genetic heterogeneity
1	100	mutated	*NRAS*	30	mutated	*NRAS*	No
2	90	mutated	*KRAS*	20	mutated	*KRAS*	No
3	95	mutated	*KRAS*	10	mutated	*KRAS*	No
4	75	mutated	*KRAS*	5	mutated	*KRAS*	No
5	95	mutated	*KRAS+PIK3CA*	30	mutated	*KRAS+PIK3CA*	No
**6**	**100**	**wild-type**		**20**	**mutated**	***BRAF***	**Yes**
**7**	**95**	**mutated**	***KRAS***	**10**	**wild-type**		**Yes**
**8**	**100**	**wild-type**		**20**	**mutated**	***KRAS***	**Yes**
**9**	**60**	**wild-type**		**10**	**mutated**	***KRAS***	**Yes**
**10**	**100**	**wild-type**		**20**	**mutated**	***KRAS + PIK3CA***	**Yes**
**11**	**100**	**wild-type**		**10**	**mutated**	***BRAF***	**Yes**
**12**	**100**	**wild-type**		**40**	**mutated**	***KRAS***	**Yes**
**13**	**100**	**mutated**	***KRAS***	**10**	**wild-type**		**Yes**
14	100	mutated	*KRAS*	20	Non amplified		NA
15	80	wild-type		0	Not done		NA
16	80	wild-type		20	wild-type		No
17	80	wild-type		10	wild-type		No
18	80	wild-type		10	wild-type		No
19	90	wild-type		20	wild-type		No
20	100	wild-type		25	wild-type		No
21	100	wild-type		15	wild-type		No
22	90	wild-type		10	wild-type		No
23	100	wild-type		30	wild-type		No
24	100	wild-type		20	wild-type		No
25	90	wild-type		20	wild-type		No
26	90	wild-type		10	wild-type		No
27	100	wild-type		20	wild-type		No
28	80	wild-type		60	wild-type		No
29	100	wild-type		5	wild-type		No
30	100	wild-type		50	wild-type		No
31	90	wild-type		20	wild-type		No

* Percentage of remaining tumour cells

** Mutation status included the relevant genes usually tested in primary colorectal tumor and CLM (i. e., *KRAS, NRAS, BRAF* and *PIK3CA*).

### Survival (Figure 1)

**Figure 1:**
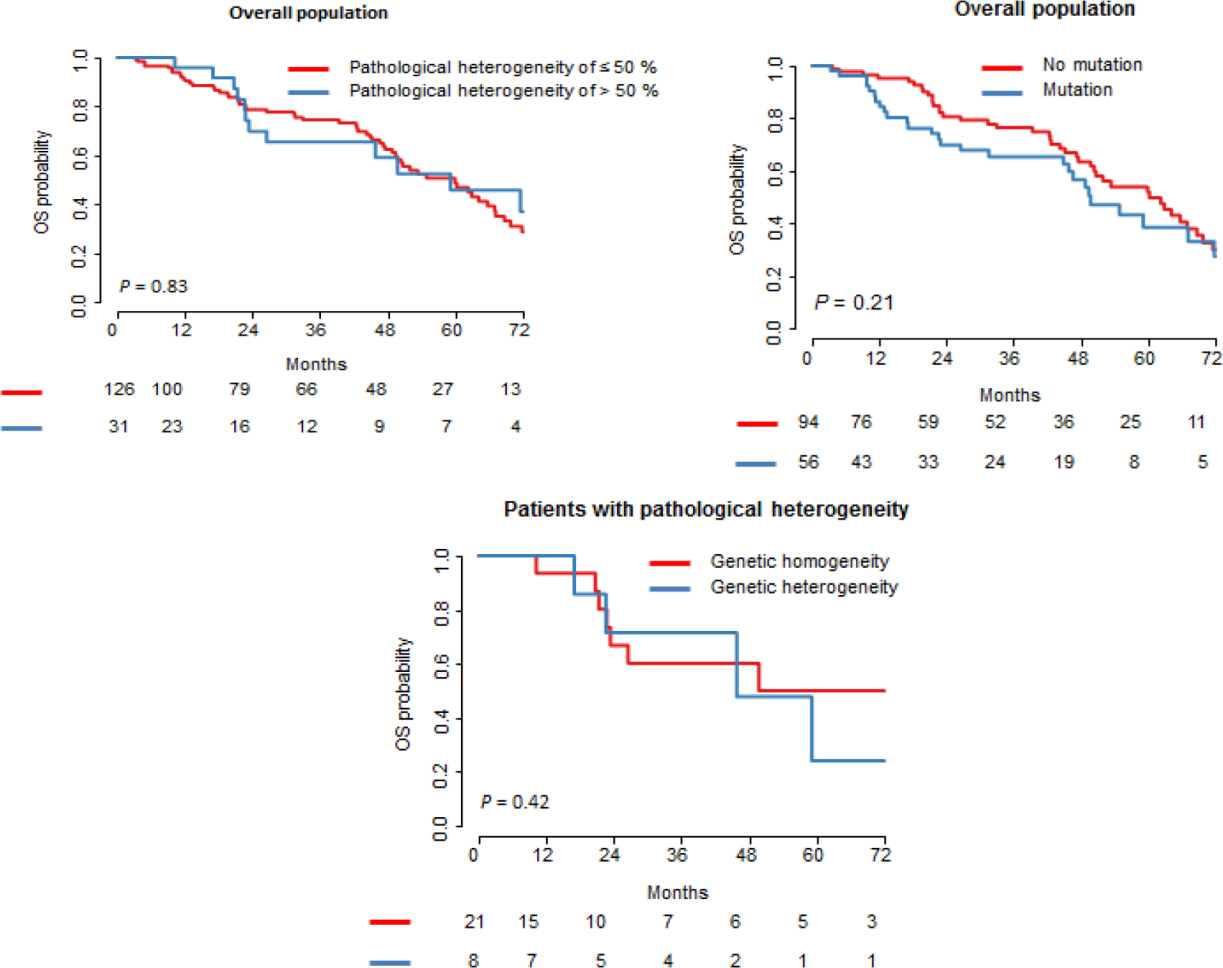
Survival curves according to the PR and genetic status There was no statistical difference in the overall survival between patients with and without pathological heterogeneity, and between patients with and without mutation. In the patients with pathological heterogeneity, there was also no difference in the overall survival between patients with and without genetic heterogeneity.

The median follow-up period was 52.8 months (range: 1–109 months) CI [48.9–68.7 months]. The cumulative 5-year overall survival rate was 48%. There was no statistical difference in the overall survival between patients with and without pathological heterogeneity, and between patients with and without mutation. In the patients with pathological heterogeneity, there was also no difference in the overall survival between patients with and without genetic heterogeneity.

## DISCUSSION

This study demonstrated a pathological heterogeneity of > 50% between the CLM in response to therapy in 19.7% of patients. The mean difference in the PR between CLM was independently associated with the use of preoperative PVE and a number of CLM > 3. A genetic heterogeneity was observed in 27.6% of the patients with pathological heterogeneity. The pathological and genetic heterogeneity did not impact the overall survival.

To date, four methods for assessing the PR to preoperative chemotherapy have been validated [3–6]. These methods based on the presence of viable cancer cells however differed in patients with multiple CLM. Rubbia-brandt et al. defined five tumour regression grades (TRG) [3]; Blazer et al. accounted for the percentage of viable cancer cells [4]. Maru et al. defined the tumour cell viability by the maximum tumour thickness at the tumour-normal interface [5]. Recently, we proposed a new method which accounted for the number of nodules, their size and their respective percentage of viable tumour cells [6]. The means of calculating the PR by the three latter methods implied that the response to chemotherapy could vary from one CLM to another. However, the pathological heterogeneity between CLM was not explicitly studied. For Rubbia-brandt et al., the morphology of CLM within the same patient was similar, with TRG being equal between nodules or within one grade range in most cases (90%). The variability of TRG between nodules in range of two grades was observed in 10% [3]. This could justify that this method accounted for one CLM (i.e., the most florid one) in contrast to the other methods. In this study, the rate of pathological heterogeneity >50% between CLM was of 19.7% (31/157), near 2-fold higher than that reported by Rubbia-brandt et al. (10%, 12/111). As for CLM, only a few prior studies focused on the comparison of PR in primary colorectal tumours, regional lymph nodes and CLM. In the study by Gervaz et al. [8], CLM exhibited a better PR than primary tumours and an identical poor response for primary tumours and lymph nodes. Two studies on patients with T3/T4 rectal cancer downstaged by neaoadjuvant chemoradiation to pT0 reported a 17% rate of positive mesorectal lymph nodes [9, 10].

Our result demonstrated that a greater heterogeneity in PR was associated with a number of CLM > 3 and the use of preoperative PVE. Because PVE is mainly used in patients with a great number of CLM, which is a significant factor of heterogeneity, a potential confounding impact of PVE could be opposed. However, under the multivariate analysis, the use of PVE and a CLM number > 3 remained independent factors for pathological heterogeneity. CLM are thought to mainly depend on arterial supply, which serves as a rational for hepatic arterial infusion strategy. The suppression of portal flow following PVE may enhance arterial flow [11], and then improve the efficacy of post PVE/ligation chemotherapy via a better drug delivery and higher concentrations of cytotoxic drugs. Thus, pathological heterogeneity would result from difference in CLM locations affected heterogeneously by embolization. In contrast, pathological heterogeneity was not statistically associated with the number of preoperative chemotherapy cycles. These results argue that the histological differences in response to chemotherapy are probably related more to the microenvironment than to tumour cell chemosensitivity.

Information not only on *KRAS* but also in *NRAS* mutational status is now an essential prerequisite for the selection of a targeted therapy based on anti-EGFR therapies. Moreover, mutations in other genes such as *BRAF* and *PIK3CA* are also associated with resistance to anti-EGFR therapies and/or worse survival [7]. A lot of studies have focused on the comparison of mutational status in primary colorectal tumours, regional lymph nodes and metastasis. In term of frequency, an association between *RAS* mutations and more aggressive tumour biology was reported: A higher incidence of gene mutation was shown in patients with primary tumours of stage III/IV compared with stage I/II [12], in patients with metachronous CLM detected after chemotherapy for the primary colorectal cancer [13–15], as well as in patients with metastases at particular sites such as lung and brain [16–18]. Because of our centre referral pattern, the gene profile was assessed in CLM and not in primary colorectal cancer. We observed a 30.6% rate of *KRAS* mutations, similar to that reported [16, 19], 2% rate of *BRAF* mutation, 2.7% rate of *NRAS* mutations and 4.7% rate of *PIK3CA* mutations and concomitant mutations of *PIK3CA* and *KRAS* occurred in 2.7% of patients. In total, 37.3% of patients showed at least one mutation.

Usually only one single sample is analysed per patient and no general recommendation exist as to which tumour sample should preferentially be tested [20]. Some studies demonstrated a high concordance between primary tumours and metastases [21–23]. Thus, the genomic profiling is usually tested in primary tumour biopsies: Material from metastatic sites is not routinely collected, generally of poorer quality and contains fewer tumour cells as a result of tumour necrosis or chemotherapy-induced changes. Other studies demonstrated discordance and thus suggested that metastatic tissues should be tested [12, 18, 24, 25]. Lymph nodes metastases were not reliable tissue specimens to define the *KRAS* mutation [12].

To our best knowledge, this study is the first to address the intermetastatic heterogeneity not only on a pathological but also genetic level within CLM in a given patient. We showed that genetic heterogeneity (one mutated CLM/one wild-type CLM) was observed in 27.6% (8/29) of the patients with pathological heterogeneity and genetic homogeneity corresponding to either any mutation (16/29, 55%) or mutations (5/29, 17.2%) within both CLM in 72.4%. In both mutated CLM, there was concordance of mutation type in 100% (5/5). Our results should lead to test additional CLM in case of wild-type results in the particular subpopulation of patients with pathological heterogeneity. Indeed, the genotyping of multiple CLM is unusual in routine practice because a such strategy is time and money consuming. This explains the lack of genotyping of all paired CLM in the 157 patients which is a limitation of our study. We also found that the mutated CLM was the less florid one in 6/8 patients (75%). This result should help pathologists in the selection of the block for the genotyping. Nevertheless, the development of liquid biopsy based on genetic testing from circulating cell free tumour DNA containing mutation in plasma has been reported as a non-invasive, specific and highly sensitive approach for monitoring disease load [26–30]. Liquid biopsies should advantageously replace tumour-section analysis although caution must be taken with respect to the short half life of the cell free DNA and its decrease during cytotoxic chemotherapy. The perspectives include the assessment of radicality of primary and secondary resections, early detection of recurrence after liver metastasectomy and monitoring of response/resistance to chemotherapy. Before surgery, it should also be a tool to solve the potential existence of a genetic heterogeneity between the primary tumour and the metastases, and between metastases since cell free DNA can be released from each CLM or all parts of the tumour.

Numerous classical factors including carcinoembryonic antigen, maximal tumor size or tumor number…, are associated with recurrence and poor survival while some recent studies have questioned their prognostic value. There is increasing evidence that the PR to preoperative chemotherapy is a major determinant of outcome after resection [3–6], independently of others prognostics factors. This study showed that pathological heterogeneity was not associated with PR. This could explain that the pathological heterogeneity did not impact the overall survival.

In conclusion, we showed an intermetastatic pathological heterogeneity in 19.7% of patients, which was independently associated with the use of PVE and a number of CLM > 3. We showed intermetastatic heterogeneity in gene expression in 27.6% of patients of the patients with pathological heterogeneity and homogeneity in type of gene mutation in 100% of patients. Existing intermetastatic heterogeneity could have clinical implications and refine guide management for tissue sampling.

## PATIENTS AND METHODS

### Study population

We included patients who were previously identified and characterized [6]. Briefly, among the 425 patients who underwent elective liver resection for CLM at Paul Brousse Hospital (Villejuif, France) between 2004 and 2011, patients with operative mortality and those with incomplete surgery (R2 resection) have been excluded. In total, 223 patients with either R0 or R1 resection who received at least 3 cycles of preoperative chemotherapy with no more than two lines in association and in whom the tissue material was available for pathologic review have been eligible for the previous study. For the purpose of the current study concerning the intermetastatic heterogeneity, the 66 patients with a single CLM were excluded from the prior study.

### Pathological evaluation

In each liver resection specimens, all CLM have been sampled entirely for CLM < 2 cm and extensively from the centre to the periphery (as recommended, one sample per cm along the greatest dimension) for bigger lesions, respectively. Formalin-fixed paraffin embedded tissue blocks were cut at 4 μm thickness and stained with haematoxylin and eosin. All stained sections have been reviewed by a single pathologist blinded to clinical information. In each CLM, the percentage of area with remaining viable tumour cells in relation to the total area of the CLM was evaluated. Pathological heterogeneity was arbitrarily defined as a difference of > 50% in the percentage of viable tumour cells between the CLM with the lower and higher response in a given patient. In each patient, the PR has been assessed according to the method by Blazer and al [4] and Sebagh et al. [6].

### Tumour DNA preparation and gene mutation profiling

In our Pathology Department, pathologists usually choose a paraffin-embedded block from the most florid CLM. Following the assessment of the percentage of tumour cells in relation with the sample area (including non tumoral liver and stroma of the tumour), the block was subsequently cut at 30μm and microdissected if containing less than 10% of tumoural cells. In patients with pathological heterogeneity, DNA extraction was performed within two samples from the most heterogeneous CLM on a pathological level. DNA extraction was performed using the QIAmp DNA Mini kit (Qiagen, Courtaboeuf, France) according to the manufacturer instructions.

All the patients were retrospectively and completely tested for the relevant genes usually tested in primary colorectal tumor and CLM (*i.e.*, *KRAS, NRAS, BRAF* and *PIK3CA*). Somatic gene mutations were detected using the MassARRAY iPLEX platform (Sequenom-Agena Bioscience, San Diego, US), which involves a three-step process consisting of the initial PCR reaction, inactivation of unincorporated nucleotides by shrimp alkaline phosphatase and a single-base primer extension. Then, the products are nano-dispensed onto a matrix-loaded silicon chip (SpectroChipII, Sequenom-Agena Bioscience, San Diego, US) and finally, the mutations are detected by MALDI-TOF (matrix-assisted laser desorption-ionization-time of flight) mass spectrometry. The experimental sensitivity of the assay was estimated to be below 5% for each gene mutation.

### Statistical analysis

Continuous data were expressed as a median (range) and/or mean (standard deviation) whereas categorical data were expressed as percentages. Categorical data were compared using Fisher's exact test or chi-square test as appropriate. Univariate and multivariate analysis were used to examine the relationship between various clinical and histological factors and 1) pathological heterogeneity (as present or absent), and 2) mean difference in the PR between the CLM with the lower and the higher response. Variables with a *P* value 0.15 under univariate analysis were included for multivariate analysis. The cumulative survival rate was calculated by the Kaplan-Meier and compared by log-rank test. Statistical significance was indicated by a *P* value < 0.05. Calculations were performed using both R (2.14.1).

## Abbreviations

CLM: colorectal metastases
PR: pathological response
TRG: tumor regression grade
PVE: portal vein embolization.
